# Effectiveness and cost-effectiveness of a progressive, individualised walking and education program for prevention of low back pain recurrence in adults: statistical analysis plan for the WalkBack randomised controlled trial

**DOI:** 10.1186/s13063-023-07119-0

**Published:** 2023-03-16

**Authors:** Natasha C. Pocovi, Petra L. Graham, Chung-Wei Christine Lin, Simon D. French, Jane Latimer, Dafna Merom, Anne Tiedemann, Christopher G. Maher, Johanna M. van Dongen, Ornella Clavisi, Mark J. Hancock

**Affiliations:** 1 Department of Health Sciences, Faculty of Medicine, Health and Human Sciences, Macquarie University, Sydney, Australia; 2 School of Mathematical and Physical Sciences, Macquarie University, Sydney, Australia; 3 The University of Sydney, Sydney Musculoskeletal Health, Gadigal Country, Sydney, Australia; 4 Department of Chiropractic, Faculty of Medicine, Health and Human Sciences, Macquarie University, Sydney, Australia; 5 School of Health Sciences, Western Sydney University, Sydney, Australia; 6 Department of Health Sciences, Vrije University of Amsterdam, Amsterdam, Netherlands; 7 Musculoskeletal Australia, Melbourne, VIC Australia

**Keywords:** Low back pain, Prevention, Walking, Randomised-controlled trial, Statistical analysis plan

## Abstract

**Background:**

Exercise for the prevention of low back pain recurrences is recommended, but under-researched. The effectiveness and cost-effectiveness of a walking program for preventing low back pain recurrence remains unknown. This a priori statistical analysis plan describes the methods of analysis for the WalkBack trial.

**Methods:**

WalkBack is a prospectively registered, pragmatic, randomised controlled trial. The aim is to investigate the effectiveness and cost-effectiveness of a 6-month progressive and individualised walking and education program (intervention) for the prevention of low back pain recurrences, compared to a no-treatment control group. The primary outcome is days to the first recurrence of an episode of activity-limiting low back pain. Key secondary outcomes include days to any recurrence of low back pain, days to a care-seeking recurrence of low back pain, disability level, health-related quality of life, costs associated with low back pain and adverse events. All participants will be followed for a minimum of 12 months. Analysis will follow the intention-to-treat principle. Cox regression is planned to assess the effects for the outcomes of time to activity-limiting, minimal and care-seeking recurrence. Hazard ratios and median survival times with 95% confidence intervals will be calculated. The effect of the intervention on continuous outcomes will be estimated with repeated-measure linear mixed models. An economic evaluation will be performed from the societal perspective for recurrence prevented (yes/no) and quality-adjusted life years. The proportion of adverse events between groups will be compared using Fisher’s exact test.

**Discussion:**

The WalkBack trial will provide evidence on the effectiveness and cost-effectiveness of a walking intervention to prevent low back pain recurrences. This statistical analysis plan provides transparency on the analysis of the trial.

**Trial registration:**

WalkBack - Effectiveness and cost-effectiveness of a progressive individualised walking and education program for the prevention of a recurrence of low back pain. ACTRN12619001134112. Date Registered: 14/08/2019.

**Supplementary Information:**

The online version contains supplementary material available at 10.1186/s13063-023-07119-0.

## Background

Low back pain (LBP) is the mostprevalent musculoskeletal condition worldwide and has been the leading cause of years lived with disability over the past three decades [1]. Isolated episodes of non-specific LBP have a favourable prognosis [2], but LBP is often recurrent with approximately 70% of individuals experiencing a new episode within 12 months following recovery [3]. Exercise appears to prevent recurrences of LBP and work-related absenteeism; however, few large-scale trials have examined scalable exercise interventions which are both accessible and affordable to recurrent LBP sufferers [4–6].

The WalkBack trial is a pragmatic, parallel-group, randomised controlled trial (RCT) investigating the effectiveness and cost-effectiveness of a progressive and individualised walking and education program compared to a no-treatment control group, in reducing recurrences of LBP. The protocol for the study has been published elsewhere and an internal protocol exists (version 4.1) [7]. Recruitment concluded in June 2022 and 701 participants were randomised to either the intervention (walking and education program) or a no-treatment control group (usual care). Provision of the intervention period (6 months) concluded in December 2022 and the final follow-up of outcome data will conclude in June 2023. Here, we describe the statistical analysis plan to guide data analyses for the trial and outline planned future supplementary analyses. Final statistical analysis will be performed following the completion of data collection, integrity checks and database lock (estimated September 2023).

### Study overview

#### Objectives

The primary objective is to determine the effectiveness of a progressive and individualised walking and education program compared to a no-treatment control group, for reducing recurrences of LBP (primary outcome). The trial hypothesis is that the participants randomised to the intervention arm will experience a greater number of days from the time of randomisation to the first self-reported recurrence of an episode of LBP (i.e. superiority). The null hypothesis is that there is no difference in days to the first self-reported recurrence between the two arms of the trial.

Secondary objectives include determining whether the walking and education program:

- Improves health-related quality of life and disability.
- Is cost-effective from a societal perspective, including data on healthcare utilisation and productivity loss (work absenteeism) due to LBP.
- Increases levels of physical activity.
- Is acceptable and adhered to, based on a series of compliance measures.
- Results in any greater risk of adverse events when compared to no treatment.

#### Study population

Participants were recruited via community advertising (e.g. via social media, company newsletters, flyer distribution) or through clinician referrals (e.g. General Practitioners (GP), physiotherapists, chiropractors, surgeons). All advertising directed people with enquiries to the trial website where potential participants could find out more about the trial, express interest, and complete a pre-screening questionnaire. Individuals identified as potentially eligible based on pre-screening then underwent full screening and explanation of the trial over the phone by a member of the research team prior to inclusion.

We recruited 701 consenting participants based on the following inclusion criteria:

- Aged 18 years and older.
- Experienced an episode of non-specific *activity-limiting LBP* within the last 6 months. Non-specific refers to pain not attributed to a specific diagnosis (e.g. vertebral fracture and cancer).
- Recovered at the time of enrolment in the trial. Recovery was defined as > 7 consecutive days with pain no greater than 1 on a 0–10 scale.

Participants were excluded at the time of screening based on the following criteria:

- Co-morbidity preventing safe participation in a walking program.
- Current participation in an exercise program aiming to prevent recurrence of LBP.
- Walking for exercise 3 or more times per week for at least 30 min per day.
- Achieving more than 150 min of moderate or vigorous intensity physical activity weekly (across a minimum of 3 days per week).
- Spinal surgery in the preceding 6 months.
- Currently pregnant.
- Inadequate English to complete outcome measures (i.e. questionnaires).

There was a change made to the exclusion criteria in response to the COVID-19 pandemic. Initially, participants were only eligible for the trial if they were able to attend face-to-face consultations with the trained study physiotherapists in Sydney, Australia. The decision was made to transition to telehealth delivery of the intervention on the 1st of April 2020, to ensure the safety of both participants and the clinicians. This was done following consultation with trial clinicians, who reported confidence in delivering the intervention via telehealth.

As COVID-related restrictions eased, participants were offered the option of telehealth or face-to-face delivery of the intervention. This change in protocol allowed for recruitment to be extended across Australia, with geographical location no longer being a barrier to enrolment. These changes were approved by the Macquarie University Ethics Committee and updates were made to the Australian New Zealand Clinical Trials Registry (ANZCTR).

#### Outcomes

For the outcomes related to LBP recurrence, participants are contacted monthly to complete an online questionnaire hosted on a REDCap (Research Electronic Data Capture) server. If the questionnaire is not completed following an email reminder, data is then collected via telephone by a research assistant blinded to group allocation, documented in a hard copy version (i.e. paper), then immediately entered in REDCap. An overview of outcomes, outcome measures, instruments and assessment time points appear in Additional file Appendix 1.

##### Primary outcome

The primary outcome for this trial is:

- *Activity-limiting LBP* *recurrence*, defined as a return of LBP lasting at least 24 h with a pain intensity > 2 (0–10 Numeric Pain Rating Scale, NPRS) with ‘activity limitation’ confirmed by a response of “somewhat” or greater on an adapted version of item PI9 of the PROMIS item bank (“How much did low back pain interfere with your day-to-day activities?” Not at all; A little bit; Somewhat; Quite a bit; Very much) [8].

##### Secondary effectiveness outcomes

- *Any LBP recurrence*is defined as a return of LBP lasting at least 24 h with a pain intensity > 2 (0–10 Numeric Pain Rating Scale). This is the lowest recurrence threshold to define any new episode of recurrence [9].
- *Care-seeking LBP recurrence is* defined as a return of LBP lasting at least 24 h with a pain intensity > 2 (0–10 Numeric Pain Rating Scale) leading to care-seeking with a healthcare provider (e.g. GP, physiotherapist, chiropractor, massage therapist, and acupuncturist).
- *Disability*will be measured by the Roland-Morris Disability Questionnaire (RMDQ) collected quarterly across 12 months [10].
- *Health-related quality of life*will be measured by the EuroQol 5-Dimension 5-Level (EQ-5D-5L) questionnarie collected quarterly across 12 months, and valued using the Canadian tariff [11, 12].

##### Health economics outcomes

These outcomes will be self-reported by participants and collected quarterly across 12 months, unless otherwise indicated:

- *Quality-adjusted life year (QALY) *will be calculated from the EQ-5D-5L utility values using the area under the curve approach [11].
- *Intervention costs* will be micro-costed by the research team (i.e. not self-reported by participants) and valued at published standard rates (e.g. Workers Compensation Rates). In addition, the cost for equipment (i.e. pedometer) and printing of resources (e.g. walking diary) will be based on accounting records of the research team.
- *Hospitalisations* will be measured by asking participants “Did you have any hospital admission in the last 3 months due to low back pain?” If so, admission date and discharge date will be recorded to calculate total stay (i.e. days). Hospitalisation will be valued using standard rates published by the Medical Benefits Scheme.
- *Healthcare services* will be measured by asking participants “Did you use any healthcare services (e.g. GP, physiotherapy, x-rays), or community health or other services (e.g. meals on wheels) in the last 3 months due to low back pain?” If so, this will be recorded as free text and frequency of use reported. The use of public health services will be valued using standard rates published by the Medical Benefits Scheme. The use of private healthcare services (e.g. physiotherapy), will be valued using standard rates published by the relevant professional body.
- *Medications* will be measured by asking participants “Did you take any prescription or over the counter medication for your low back pain in the last 3 months?” If so, medication name, administration route (e.g. by mouth and applied to skin), strength, dosage and days utilised, will be recorded as free text. Medication use will be valued using standard rates published by the Pharmaceutical Benefits Scheme.
- *Work absenteeism* will be measured by asking participants “Did you miss any hours off your normal paid work in the last 3 months due to low back pain?” If so, this will be recorded as free text and the frequency reported. Costs will be estimated by the number of days absent from work multiplied by the average wage rate as derived from the Australian Bureau of Statistics.
- *Societal costs* will include the costs of the intervention, hospitalisations, other healthcare services, medications, and work absenteeism.

##### Physical activity outcomes

Daily walking will be measured via a hip-worn triaxial accelerometer (Actigraph GTX3), proven accurate and valid for quantifying physical activity [13]. All participants, regardless of treatment group allocation, will be instructed to wear the activity monitor for a 7-day window, 3 months following enrolment to the trial. This outcome will be collected via an objective data source to demonstrate any potential differences in physical activity between the intervention and control groups following 3 months of involvement in the walking intervention.

To source valid physical activity data, we will identify a valid wear day as ≥ 10 h and include participants with at least 4 valid wear days, which is the minimum number of days needed to reliably estimate free-living physical activity [14]. We are specifically interested in the following physical activity parameters:

- *Steps per day*. Steps per day will be averaged across the available valid days, for a minimum of 4 days and a maximum wear time of 7 days.
- *Brisk steps per day*. Brisk walking will be defined as a cadence > 100 steps per minute in accordance with previous literature [15]. Brisk steps per day will be averaged across the available valid days, for a minimum of 4 days and a maximum wear time of 7 days.
- *Minutes of moderate to vigorous physical activity (MVPA).*Time spent in activity of a defined intensity (MVPA) will be determined by summing minutes in a day where the count met the pre-set criterion based on previous literature and recommendations from the ActiGraph Group [14]. Mean daily time in MVPA will be averaged across the available valid days, for a minimum of 4 days and a maximum wear time of 7 days.

A self-report measure of physical activity will also be collected from all participants (intervention and control), using a modified version of the International Physical Activity Questionnaire – Short Form (IPAQ-SF) [16].

##### Adverse events outcomes

Adverse event data will be self-reported and collected at quarterly intervals (months 3, 6, 9 and 12). The following definitions will be used:

- *Adverse event*, any untoward medical occurrence in a participant that does not necessarily have a causal relationship with this treatment.
- *Serious adverse event*, any adverse event that resulted in death, was life-threatening, required hospitalisation, or resulted in persistent or significant disability or incapacity. These events do not necessarily have a causal relationship with the treatment.

Adverse events and serious adverse events will be coded according to the International Classification of Diseases (ICD-11) using three-digit codes [17].

##### Study adherence outcomes

Various measures of compliance will be collected throughout the trial. We will specifically collect the following measures of adherence:

- *Session attendance*, measuring attendance to the intervention consultations as documented by the treating physiotherapist.
- *Intentional walking duration*, collected via a participant-completed walking diary. Those allocated to the intervention will be asked to maintain a walking diary for the first 3 months of the intervention, documenting the duration of intentional walking (as opposed to incidental walking) in minutes.
- *Self-reported adherence*, measured using the modified version of the Brief Adherence Rating Scale (BARS) [18]. In this rating scale, participants allocated to the intervention will be asked to rate their adherence to the prescribed walking program from 0 (not compliant at all) to 10 (very compliant) at quarterly intervals (months 3, 6, 9 and 12).

##### Co-intervention outcomes

The use of any co-interventions received by participants will be monitored throughout the trial and reported by patients. This will be collected at quarterly intervals (months 3, 6, 9 and 12) using the following question: “Apart from your involvement in the study have you received any additional treatment or prevention approach for back pain over the last 3 months?”.

#### Sample size and randomisation

A sample size calculation indicated that 349 participants per group (698 total) would provide 80% power to detect a 25% relative reduction in recurrence rates of *activity-limiting LBP* (i.e. primary outcome) in the intervention group, compared to the control group. We used a conservative estimate of 30% recurrence rate at 12 months in the control group, a rate observed in previous work by Stanton et al. [19] A 25% relative reduction (from 30 to 22.5%) is large enough to have important public health implications for such a simple intervention. Specifications include a two-sided log-rank test, Type-I error = 0.05, 24-month accrual period, and 12-month follow-up period. Sample size calculations allowed for 1% loss to follow-up per month.

Randomisation to intervention or control was conducted using a pre-generated schedule and allocation concealment was ensured at the patient level. Randomisation used randomly permuted blocks of 4, 6 and 8 and participants were stratified by history of > 2 previous lifetime episodes of LBP (known to be a prognostic factor for recurrence) [3], and recruitment from the community versus clinician referral.

#### Blinding

Due to the nature of the intervention, it was not possible to blind participants or intervention providers to group allocation. However, the researchers responsible for follow-up data collection and statistical analysis are blinded to group allocation. Interpretation of results will occur based on masked review of data (i.e. non-disclosure of which data comes from the intervention or control group). Once consensus among authors is reached regarding the interpretation of the masked results, the randomisation code will be broken, and the manuscript finalised to reduce interpretation bias [20].

#### Intervention

Participants allocated to the intervention group receive an individualised and progressive walking and education program, provided by registered physiotherapists trained for the trial, from 25 private practice sites across the states of New South Wales and Queensland in Australia. Initial plans assumed the intervention would involve six sessions comprising an even split of face-to-face and telehealth consultations for all participants. Due to COVID-19, and government advice in Australia related to the pandemic, temporary restrictions were placed on the provision of face-to-face consultations and so some patients received all elements of the intervention via telehealth. The broad aim of these sessions is to design a progressive and individually tailored walking program with each participant, targeting a dosage of walking of 5 times per week, for at least 30 min by 3 months into the intervention.

The delivery of the intervention has been framed around the principles of health coaching, with the intention of supporting behaviour change and optimising compliance. Another method to facilitate compliance is the provision of a pedometer and walking diary to act as motivators in completing the program and to provide participants with a degree of accountability. The education component of the intervention focuses on a modern understanding of LBP, aimed to reduce the threat and fear associated with pain, alongside advice related to strategies to reduce the risk of a recurrence of LBP. Participants in the control group receive no intervention as part of their involvement in the trial. Detailed information related to the intervention and control groups can be found in the published protocol [7].

### Statistical analysis

#### Analysis principles

All analyses will be conducted using R software (version 4.2.2 or above) or STATA (version 17.0 or above). Analyses of the primary and secondary outcomes will be conducted by a statistician (member of the trial team), taking no part in participant recruitment or data collection. Analyses will follow intention-to-treat principles, with all participants analysed according to their randomised allocation. A masked sample of the data will first be used to trial and confirm statistical procedures. Next, the fully masked dataset will be used, with data interpretation occurring prior to unblinding of group allocation. There is no intention to conduct interim analyses.

Categorical variables will be summarised by frequencies and percentages. Continuous variables will be reported using standard measures of central tendency and dispersion, either mean and standard deviation (SD) or median and interquartile range, depending on the shape or skewness of the data. A *p*-value < 0.05 will be considered statistically significant. A 95% confidence interval (CI) will be reported to aid the interpretation of precision and the clinical importance of findings.

#### Participant flow

**Fig. 1:**
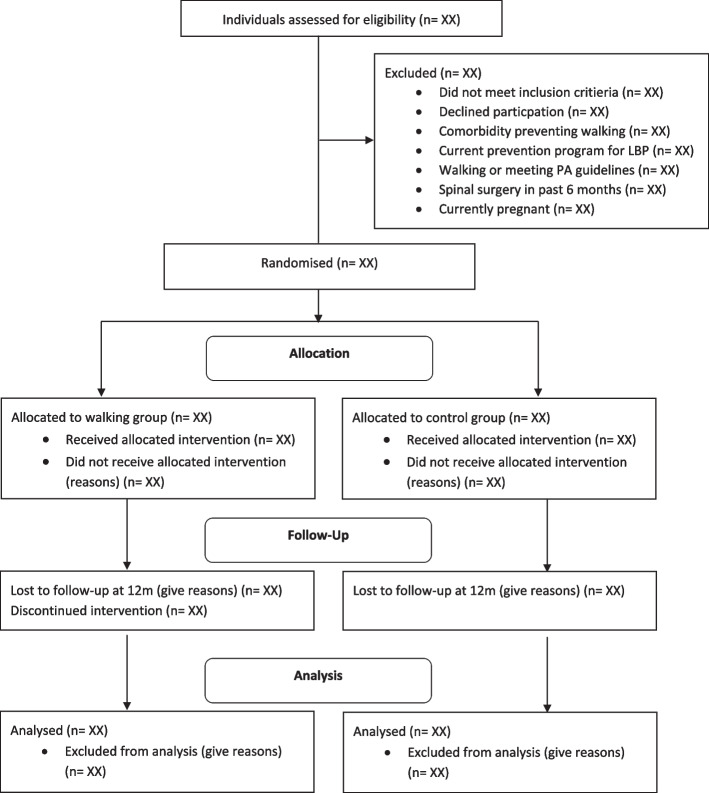
Consort flow diagram

A flow diagram presented in accordance with the CONSORT template (Fig. 1), will be included to identify participation, withdrawal, and loss to follow-up throughout the trial. Reasons for withdrawal will also be presented in the flow diagram. We will report the number and proportion of participants successfully followed up until either experiencing the primary outcome or reaching the final follow-up at 12 months.

#### Baseline participant characteristics

Baseline characteristics will be presented separately for each arm of the trial. Data collected from the baseline questionnaire including age, gender, work and educational status, and relevant history of back pain will be presented in a table. The full list of baseline characteristics and demographics that will be reported is presented in Table 1.

**Table 1:** Baseline characteristics of participants

	**Intervention (*n* = xxx)**	**Control (*n* = xxx)**
Female	*n*/*N* (%)	*n*/*N* (%)
Age (years)	xx.x (SD), *n*	xx.x (SD), *n*
BMI (kg/m^2^)	xx.x (SD), *n*	xx.x (SD), *n*
Education level		
Some secondary school	*n*/*N* (%)	*n*/*N* (%)
Completed secondary school	*n*/*N* (%)	*n*/*N* (%)
Some additional training	*n*/*N* (%)	*n*/*N* (%)
Undergraduate university	*n*/*N* (%)	*n*/*N* (%)
Postgraduate university	*n*/*N* (%)	*n*/*N* (%)
Current work status		
Full-time	*n*/*N* (%)	*n*/*N* (%)
Part time	*n*/*N* (%)	*n*/*N* (%)
Unemployed	*n*/*N* (%)	*n*/*N* (%)
Students or homeworkers	*n*/*N* (%)	*n*/*N* (%)
Sick leave or pension	*n*/*N* (%)	*n*/*N* (%)
Retired	*n*/*N* (%)	*n*/*N* (%)
Other	*n*/*N* (%)	*n*/*N* (%)
Gross weekly household income (annual in brackets)		
No income	*n*/*N* (%)	*n*/*N* (%)
$1–$649 ($1–$33,799)	*n*/*N* (%)	*n*/*N* (%)
$650–$1699 ($33,800–$88,399)	*n*/*N* (%)	*n*/*N* (%)
$1700–$3999 ($88,400–$207,999)	*n*/*N* (%)	*n*/*N* (%)
$4000 or more ($208,000 or more)	*n*/*N* (%)	*n*/*N* (%)
Smoking status		
Never	*n*/*N* (%)	*n*/*N* (%)
Current smoker	*n*/*N* (%)	*n*/*N* (%)
Ex-smoker	*n*/*N* (%)	*n*/*N* (%)
Manual task involving heavy loads		
Very frequently	*n*/*N* (%)	*n*/*N* (%)
Frequently	*n*/*N* (%)	*n*/*N* (%)
Occasionally	*n*/*N* (%)	*n*/*N* (%)
Rarely	*n*/*N* (%)	*n*/*N* (%)
Very rarely	*n*/*N* (%)	*n*/*N* (%)
Never	*n*/*N* (%)	*n*/*N* (%)
Manual task involving awkward positions		
Very frequently	*n*/*N* (%)	*n*/*N* (%)
Frequently	*n*/*N* (%)	*n*/*N* (%)
Occasionally	*n*/*N* (%)	*n*/*N* (%)
Rarely	*n*/*N* (%)	*n*/*N* (%)
Very rarely	*n*/*N* (%)	*n*/*N* (%)
Never	*n*/*N* (%)	*n*/*N* (%)
General health		
Excellent	*n*/*N* (%)	*n*/*N* (%)
Very good	*n*/*N* (%)	*n*/*N* (%)
Good	*n*/*N* (%)	*n*/*N* (%)
Fair	*n*/*N* (%)	*n*/*N* (%)
Poor	*n*/*N* (%)	*n*/*N* (%)
Low back pain		
Previous episodes	xx.x (SD), *n*	xx.x (SD), *n*
Duration of last episode (days)	xx.x (SD), *n*	xx.x (SD), *n*
Time since last episode (days)	xx.x (SD), *n*	xx.x (SD), *n*
Perceived risk of recurrence (0 to 10)	xx.x (SD), *n*	xx.x (SD), *n*
Levels of physical activity (IPAQ)		
Walking (min/week)	xx.x (SD), *n*	xx.x (SD), *n*
Moderate (min/week)	xx.x (SD), *n*	xx.x (SD), *n*
Vigorous (min/week)	xx.x (SD), *n*	xx.x (SD), *n*
Time sitting (min in average weekday)	xx.x (SD), *n*	xx.x (SD), *n*
DASS-21 (0 to 21)		
Depression	xx.x (SD), *n*	xx.x (SD), *n*
Anxiety	xx.x (SD), *n*	xx.x (SD), *n*
Stress	xx.x (SD), *n*	xx.x (SD), *n*
Sleep quality		
Very good	xx.x (SD), *n*	xx.x (SD), *n*
Fairly good	xx.x (SD), *n*	xx.x (SD), *n*
Fairly bad	xx.x (SD), *n*	xx.x (SD), *n*
Very bad	xx.x (SD), *n*	xx.x (SD), *n*
Health Status (Euro-QOL)		
EQ-5D-5L Health State Index	xx.x (SD), *n*	xx.x (SD), *n*
EQ-VAS score (0–100)	xx.x (SD), *n*	xx.x (SD), *n*

*BMI* Body mass index, *DASS-21* Depressions Anxiety Stress Scale, *EQ-5D-5L* EuroQol 5-Dimension 5-Level, *IPAQ* International Physical Activity Questionnaire, *VAS* Visual analogue scale, *QOL* Quality Of Life, *kg* kilogram, *m* metre, *SD* Standard Deviation

#### Primary analysis

Cox regression is planned to assess the effect of the treatment group on Hazard Ratios (HR) and to adjust for prognostic factors for recurrence of LBP, if these are unbalanced between groups despite randomisation. Kaplan–Meier survival plots will be constructed to visually display survival curves, with patients censored on the day of their last successful follow-up, or when the participant was identified to have the event (i.e. recurrence), whichever occurs first. HR and median survival times with 95% CIs will be reported. The effect of including the variables that were used for the stratified randomisation as strata in these analyses will also be assessed (i.e. clinician versus community referral and the number of previous episodes (up to 2 versus > 2 episodes)).

#### Secondary analyses

##### Effectiveness outcome analyses

For the outcomes of *any LBP recurrence* or a *care-seeking recurrence of LBP*, an analogous survival analysis to that of the primary outcome detailed above will be conducted.

To test the effects of the intervention on continuous secondary outcomes, between-group comparisons will be conducted using multiple linear mixed-effects regression combining all available post-randomisation measurements (at either months 3, 6, 9 or 12 depending on outcome collection). Transformations will be applied to meet model assumptions, if needed. Linear mixed modelling will be used to take account of the correlation between measurements within an individual for repeated measurements over time. A template has been provided for how the data will be reported (see Table 2).

**Table 2:** Secondary outcomes

	**Intervention (*n* = xxx)**	**Control (*n* = xxx)**	**Mean difference (95% CI), *p*-value**
Disability—RMDQ			
Month 3	xx.x (SD), *n*	xx.x (SD), *n*	xx.x (xx.x to xx.x),* p* = 0.xxx
Month 6	xx.x (SD), *n*	xx.x (SD), *n*	xx.x (xx.x to xx.x),* p* = 0.xxx
Month 9	xx.x (SD), *n*	xx.x (SD), *n*	xx.x (xx.x to xx.x),* p* = 0.xxx
Month 12	xx.x (SD), *n*	xx.x (SD), *n*	xx.x (xx.x to xx.x),* p* = 0.xxx
Health-related QoL – EQ-5D-5L			
Month 3	xx.x (SD), *n*	xx.x (SD), *n*	xx.x (xx.x to xx.x),* p* = 0.xxx
Month 6	xx.x (SD), *n*	xx.x (SD), *n*	xx.x (xx.x to xx.x),* p* = 0.xxx
Month 9	xx.x (SD), *n*	xx.x (SD), *n*	xx.x (xx.x to xx.x),* p* = 0.xxx
Month 12	xx.x (SD), *n*	xx.x (SD), *n*	xx.x (xx.x to xx.x),* p* = 0.xxx
Self-reported Physical Activity—IPAQ			
Month 3	xx.x (SD), *n*	xx.x (SD), *n*	xx.x (xx.x to xx.x),* p* = 0.xxx
Month 12	xx.x (SD), *n*	xx.x (SD), *n*	xx.x (xx.x to xx.x),* p* = 0.xxx
ActiGraph measure at 3 months^a^			
Steps per day	xx.x (SD), *n*	xx.x (SD), *n*	xx.x (xx.x to xx.x),* p* = 0.xxx
Brisk Steps per day^b^	xx.x (SD), *n*	xx.x (SD), *n*	xx.x (xx.x to xx.x),* p* = 0.xxx
MVPA minutes per day	xx.x (SD), *n*	xx.x (SD), *n*	xx.x (xx.x to xx.x),* p* = 0.xxx

*EQ-5D-5L* EuroQol 5-Dimension 5-Level, *IPAQ* International Physical Activity Questionnaire, *MVPA* Moderate-vigorous physical activity, *RMDQ* Roland-Morris Disability Questionnaire

^a^Data in above table represents a minimum 4-day, and maximum of 7-day wear period. Only data for participants whose dataset met the minimum wear time requirement of 10 h/day are included

^b^Brisk walking is defined as a cadence > 100 steps per minute

##### Economic evaluation analyses

The economic evaluation will be conducted from the societal perspective as the primary analysis, whilst the Australian health system perspective will be used for sensitivity analyses. The time horizon of the economic analysis is 12 months.

For the economic evaluation, missing cost and effect data will be imputed using multivariate imputation by chained equations with predictive mean matching [21]. The imputation model will include variables differing between groups at baseline as well as those predicting the missingness of follow-up cost and effect data. Cost and effect differences will be estimated using seemingly unrelated regression analyses, in which two regression equations are modelled simultaneously and cost and effect differences are adjusted for their possible correlations through correlated error terms. Cost and effect differences will be adjusted using the same covariates as the primary effectiveness analyses, in addition to work status and baseline utility values for QALYs [22]. To handle the right skewed nature of cost data, uncertainty surrounding cost differences will be estimated using bias-corrected and accelerated bootstrapping (5000 replications) [23].

Incremental cost-effectiveness ratios (ICERs) will be calculated by dividing the between-group difference in costs by the between-group difference in effects (i.e. cost per recurrence prevented, and cost per QALY gained). The uncertainty surrounding ICERs will be estimated using bootstrapping as well (5000 replications) and graphically presented on cost-effectiveness planes. Cost-effectiveness acceptability curves will be estimated to show the probability of the intervention being cost-effective for a range of willingness to pay (i.e. maximum amount decision-makers are willing to pay per unit of effect gained). Sensitivity analyses will be performed to assess the robustness of the results based on a complete-case analysis and an analysis from the Australian health system perspective.

##### Physical activity data analyses

To test the effects of the intervention on physical activity, between-group comparisons will be conducted using linear regression at timepoints at which each set of data will be collected (i.e. month 3 for Actigraph data; and at baseline, month 3 and month 12 for the International Physical Activity Questionnaire (IPAQ) data). Transformations will be applied to meet model assumptions, if needed. A template has been provided for how the data will be reported (see Table 2).

##### Adverse event analyses

For the measures of adverse events, we will represent the frequency and proportion of participants who experienced an adverse event, and these will be compared between groups using Fisher’s exact test as we expect the incidence of adverse events to be low (see Table 3).

**Table 3:** Adverse and serious adverse events

	**Intervention group (*n* = xxx)**	**Control group (*n* = xxx)**	***p*-value**
Serious adverse events			
Total	nEVT/nPAT (%)	nEVT/nPAT (%)	*p* = 0.xxx
Adverse event A^a^	nEVT/nPAT (%)	nEVT/nPAT (%)	
Adverse event B^a^	nEVT/nPAT (%)	nEVT/nPAT (%)	
Adverse event C^a^	nEVT/nPAT (%)	nEVT/nPAT (%)	
Etc.^a^	nEVT/nPAT (%)	nEVT/nPAT (%)	
Adverse events			
Total	nEVT/nPAT (%)	nEVT/nPAT (%)	*p* = 0.xxx
Adverse event A^a^	nEVT/nPAT (%)	nEVT/nPAT (%)	
Adverse event B^a^	nEVT/nPAT (%)	nEVT/nPAT (%)	
Adverse event C^a^	nEVT/nPAT (%)	nEVT/nPAT (%)	
Etc.^a^	nEVT/nPAT (%)	nEVT/nPAT (%)	

^a^Example categories which will be revised based on observation of final free-text data responses

##### Handling of missing data

A completeness index will be used to calculate the completeness of primary survival data [24]. Participants who are lost to follow-up will be censored on the day they last provided data, and those with no available outcome data will be censored at day one. All other available primary data will be used until recurrence (i.e. primary outcome) or reaching the end of the data collection period (i.e. minimum 12 months).

For the remaining secondary effectiveness outcomes, the number of missing observations will be reported at each relevant timepoint (i.e. months 3, 6, 9 and 12). In cases where more than 10% of the outcome data is missing, multiple imputations will be used to conduct sensitivity analyses. The need to impute will be confirmed at the time of the masked review of the data.

##### Sub-group analyses

We intend to perform exploratory sub-group analyses to assess the effect of a small number of participant baseline variables as treatment effect modifiers. Example variables considered for moderation analyses include the number of previous LBP episodes, baseline levels of physical activity, sedentary behaviour, and age. We intend to later publish a publicly available protocol (i.e. on open science framework) for subgroup analyses which will include hypotheses on the direction of effect. We anticipate only using the primary outcome for these analyses, but this may change if for example we find no main effect for the primary outcome but a worthwhile effect for one of our key secondary outcomes (e.g. disability). We also intend to conduct a Complier Average Causal Effect (CACE) analysis to estimate the effect of the intervention in compliers [25–27]. We will use a propensity score and/or a joint-modelling approach.

## Conclusion

The WalkBack trial is the first RCT exploring the use of a progressive, individualised walking and education intervention for the prevention of LBP recurrences. This manuscript details the statistical analyses planned for the trial, aiming to improve the transparency of reporting and minimise data-driven reporting of results. Any deviations from the protocol and analysis plan will be reported in the final published report.

## Supplementary Information

**Additional file 1: Appendix 1.** Overview of outcomes, outcome measures, instruments and assessment time points.

## Data Availability

As stipulated in our Data Management Plan (v1.1), the deidentified data and statistical code will be made available on request soon after each report of the data has been published. Different aspects of the data will be published separately, which will determine when those data are publicly available. A data-sharing agreement will require a commitment to using the data only for specified research purposes, to securing the data appropriately and to destroying the data after a nominated period.

## Abbreviations

ANZCTR: Australian New Zealand Clinical Trials Registry
BARS: Brief Adherence Rating Scale
CACE: Complier Average Causal Effect
CI: Confidence Interval
EQ-5D-5L: EuroQol 5-Dimension 5-Level
GP: General Practitioner
HR: Hazard Ratio
ICD: International Classification of Diseases
ICER: Incremental Cost-Effectiveness Ratio
IPAQ-SF: International Physical Activity Questionnaire-Short Form
LBP: Low Back Pain
MVPA: Moderate to vigorous physical activity
NPRS: Numeric Pain Rating Scale
PROMIS: Patient-Reported Outcomes Measurement Information System
QALY: Quality-Adjusted Life Year
RCT: Randomised Controlled Trial
REDCap: Research Electronic Data Capture
RMDQ: Roland-Morris Disability Questionnaire
SD: Standard Deviation

## References

[1] James SL, Abate D, Abate KH (2018). Global, regional, and national incidence, prevalence, and years lived with disability for 354 diseases and injuries for 195 countries and territories, 1990–2017: a systematic analysis for the Global Burden of Disease Study 2017. The Lancet.

[2] Downie AS, Hancock MJ, Rzewuska M (2016). Trajectories of acute low back pain: a latent class growth analysis. Pain.

[3] da Silva T, Mills K, Brown BT (2019). Recurrence of low back pain is common: a prospective inception cohort study. J Physiother.

[4] Steffens D, Maher CG, Pereira LS (2016). Prevention of low back pain: a systematic review and meta-analysis. JAMA Intern Med.

[5] de Campos TF, Maher CG, Fuller JT (2021). Prevention strategies to reduce future impact of low back pain: a systematic review and meta-analysis. Br J Sports Med.

[6] Huang R, Ning J, Chuter VH (2020). Exercise alone and exercise combined with education both prevent episodes of low back pain and related absenteeism: systematic review and network meta-analysis of randomised controlled trials (RCTs) aimed at preventing back pain. Br J Sports Med.

[7] Pocovi NC, Lin CWC, Latimer J (2020). Effectiveness and cost-effectiveness of a progressive, individualised walking and education programme for prevention of low back pain recurrence in adults: study protocol for the WalkBack randomised controlled trial. BMJ Open..

[8] Amtmann D, Cook KF, Jensen MP (2010). Development of a PROMIS item bank to measure pain interference. Pain.

[9] Stanton TR, Latimer J, Maher CG (2011). A modified Delphi approach to standardize low back pain recurrence terminology. Eur Spine J.

[10] Roland M, Morris RJS (1983). A study of the natural history of back pain: part I development of a reliable and sensitive measure of disability in low-back pain. Spine (Phila Pa 1976).

[11] Herdman M, Gudex C, Lloyd A (2011). Development and preliminary testing of the new five-level version of EQ-5D (EQ-5D-5L). Qual Life Res.

[12] Xie F, Pullenayegum E, Gaebel K (2016). A time trade-off-derived value set of the EQ-5D-5L for Canada. Med Care.

[13] Berlin JE, Storti KL, Brach JS (2006). Using activity monitors to measure physical activity in free-living conditions. Phys Ther.

[14] Troiano RP, Berrigan D, Dodd KW (2008). Physical activity in the United States measured by accelerometer. Med Sci Sports Exerc.

[15] Tudor-Locke C, Ducharme SW, Aguiar EJ (2020). Walking cadence (steps/min) and intensity in 41 to 60-year-old adults: the CADENCE-adults study. Int J Behav Nutr Phys Act.

[16] Lee PH, Macfarlane DJ, Lam TH (2011). Validity of the International Physical Activity Questionnaire Short Form (IPAQ-SF): a systematic review. The international journal of behavioral nutrition and physical activity.

[17] International Classification of Diseases Eleventh Revision (ICD-11). Geneva: World Health Organization; 2022. License: CC BY-ND 3.0 IGO. https://icdcdn.who.int/icd11referenceguide/en/html/index.html#copyright-page.

[18] Byerly MJ, Nakonezny PA, Rush AJ (2008). The Brief Adherence Rating Scale (BARS) validated against electronic monitoring in assessing the antipsychotic medication adherence of outpatients with schizophrenia and schizoaffective disorder. Schizophr Res.

[19] Stanton TR, Henschke N, Maher CG (2008). After an episode of acute low back pain, recurrence is unpredictable and not as common as previously thought. Spine.

[20] Järvinen TL, Sihvonen R, Bhandari M (2014). Blinded interpretation of study results can feasibly and effectively diminish interpretation bias. J Clin Epidemiol.

[21] White IR, Royston P, Wood AM (2011). Multiple imputation using chained equations: issues and guidance for practice. Stat Med.

[22] Manca A, Hawkins N, Sculpher MJ (2005). Estimating mean QALYs in trial-based cost-effectiveness analysis: the importance of controlling for baseline utility. Health Econ.

[23] El Alili M, van Dongen JM, Esser JL (2022). A scoping review of statistical methods for trial-based economic evaluations: the current state of play. Health Econ.

[24] Clark TG, Altman DG, De Stavola BL (2002). Quantification of the completeness of follow-up. The Lancet.

[25] Kasza J (2021). Research Note: estimating the complier average causal effect when participants in randomised trials depart from allocated treatment. J Physiother.

[26] Peugh JL, Strotman D, McGrady M (2017). Beyond intent to treat (ITT): a complier average causal effect (CACE) estimation primer. J Sch Psychol.

[27] Diaz Ordaz K, Franchini A, Grieve R (2018). Methods for estimating complier average causal effects for cost-effectiveness analysis. J R Stat Soc A Stat Soc.

