# Supplementary material for: Effectiveness and cost-effectiveness of a progressive, individualised walking and education program for prevention of low back pain recurrence in adults: statistical analysis plan for the WalkBack randomised controlled trial
Source: Trials. 2023 Mar 16;24:197. doi: 10.1186/s13063-023-07119-0 (PMC10019396; doi:10.1186/s13063-023-07119-0)
Supplement: Supplementary file 1 — Additional file 1: Appendix 1. Overview of outcomes, outcome measures, instruments and assessment time points. [file 13063_2023_7119_MOESM1_ESM.docx]

| Appendix 1: Overview of outcomes, outcome measures, instruments and assessment time points | | | |
| --- | --- | --- | --- |
| Outcomes | Outcome measures | Instrument | Assessment time point^a^ |
| **Primary Outcome** |  |  |  |
| Activity Limiting LBP episode | Number of days from randomisation until recurrence of an episode of LBP causing activity limitation. | Self-report Adapted version of item PI9 of the PROMIS item bank | Monthly |
| **Secondary Outcome** |  |  |  |
| Any LBP episode | Number of days from randomisation until recurrence of an episode of LBP >2/10. | Self-report | Monthly |
| Care seeking LBP episode | Number of days from randomisation until recurrence of an episode of LBP resulting in care seeking. | Self-report | Monthly |
| Health-related quality of life |  | EQ-5D-5L | T0, T1, T2, T3, T4 |
| Disability |  | RMDQ | T1, T2, T3, T4 |
|  |  |  |  |
| Health economics’ related outcomes |  |  |  |
| Health-related quality of life | Used to calculate QALYs | EQ-5D-5L | T0, T1, T2, T3, T4 |
| Hospitalisation |  | Self-report | T0, T1, T2, T3, T4 |
| Healthcare services used | Care and services used (e.g., community care, cleaning services) | Self-report | T0, T1, T2, T3, T4 |
| Medication use | Medication specifically used for the management of LBP | Self-report | T0, T1, T2, T3, T4 |
| Work absenteeism | Hours of missed work attributable to LBP | Self-report | T0, T1, T2, T3, T4 |
|  |  |  |  |
| Physical activity | Physical activity counts (7 days) | ActiGraph | T2 |
|  | Time in sitting, walking, moderate and vigorous physical activity | IPAQ-SF | T0, T1, T4 |
|  |  |  |  |
| Adverse Events |  | Self-report | T1, T2, T3, T4 |
|  |  |  |  |
| Compliance Measure |  |  |  |
| Attendance Recordb |  | Clinician Reported | T2 |
| Adherence Ratingb |  | BARS (self-report) | T1, T2, T3, T4 |
| Intentional Walkingb | Minutes reported for intentional walking for exercise | Walking Diary | 0-3 months |
|  |  |  |  |
| Co-interventions received | Additional treatment or prevention sought for LBP management | Self-report | T1, T2, T3, T4 |

aAssessment time points: T0 = baseline pre-intervention, T1 = month 3 post intervention, T2 = month 6 post intervention, T3= month 9 post intervention, T4 = month 12 post intervention.

bCompleted by Intervention Group only.

*BARS, Brief Adherence Rating Scale; EQ-5D-5L, EuroQol 5-Dimension 5-Level; IPAQ-SF, International Physical Activity Questionnaire-Short Form; LBP, low back pain; PROMIS, Patient-Reported Outcomes Measurement Information System; QALY, Quality Adjusted Life Year; RMDQ, Roland-Morris Disability Questionnaire.*
